# Increasing pH leads to more efficient production of polyhydroxyalkanoates from volatile fatty acids using *Cupriavidus necator H16*

**DOI:** 10.1186/s12934-026-03135-2

**Published:** 2026-10-05

**Authors:** Steven Leonhardt, Birk Achenbach, Pravesh Tamang, Günter E. M. Tovar, Susanne Zibek

**Affiliations:** 1 Fraunhofer Institute for Interfacial Engineering and Biotechnology IGB, Nobelstr. 12, 70569 Stuttgart, Germany; 2 Institute of Interfacial Process Engineering and Plasma Technology IGVP, University of Stuttgart, Pfaffenwaldring 31, 70569 Stuttgart, Germany; 3 Present Address: Institute of Bioprocess Engineering and Pharmaceutical Technology IBPT, TH Mittelhessen University of Applied Sciences, Stuttgart, Germany; 4 Institute of Bioprocess Engineering and Pharmaceutical Technology IBPT, TH Mittelhessen University of Applied Sciences, Stuttgart, Germany

**Keywords:** Cupriavidus necator, Volatile fatty acids, VFA, pH, Weak acid toxicity, PHA, Fed-batch

## Abstract

Volatile fatty acids (VFAs) are increasingly recognized as valuable feedstocks for the sustainable production of industrial chemicals in the context of a sustainable bioeconomy. However, their weak acidic character contributes to microbial toxicity. At lower pH values, VFAs predominantly occur in their undissociated form, which can permeate the cell membrane, disturb intracellular metabolism and increase the cellular energy burden. In this study, we investigated the effect of pH on microbial growth and polyhydroxyalkanoate (PHA) production in *Cupriavidus necator* H16 cultivated on a VFA mixture.

To determine the influence of VFA concentrations on growth behaviour, batch experiments were conducted in microbioreactors using different concentrations of a VFA mixture. These experiments revealed multiphasic growth at VFA concentrations > 2.0 g∙L^− 1^. Consequently, all subsequent fed-batch experiments were conducted at concentrations below this threshold.

To assess pH-dependent effects on growth and PHA production, fed batch experiments were carried out in a 1 L bioreactor at pH values between 6 and 8.5 using a pH-pO_2_-stat feeding strategy. Optimal growth performance was achieved at pH 7.5, as indicated by the highest specific growth rate (*µ*_*App*_ = 0.21 ± 0.01 h^− 1^) and biomass yield on VFA (*Y*_*X/VFA*_ = 0.58 ± 0.01 g∙g^− 1^). Compared to the commonly used pH of 7.0, these results correspond to a 31% increase in the specific growth rate and a 24% increase in the biomass yield c. In contrast, optimal PHA production was observed at pH 8.0, resulting in a PHA concentration of 42.5 g∙L^− 1^ and a PHA yield on VFA of 0.53 ± 0.03 g∙g^− 1^. Thus, growth rates, biomass yields, and PHA yields decreased at pH values above 8.0, while no growth at all was detected at pH 8.5.

These obtained results demonstrate a strong influence of pH on VFA utilization, growth performance, and PHA biosynthesis by *C. necator H16*. Based on the observed responses, pH 7.5 is recommended for biomass production, whereas pH 8.0 is optimal for PHA synthesis when VFAs are used as the sole carbon source.

## Introduction

 Polyhydroxyalkanoates (PHAs) are a promising class of biodegradable microbial polyesters with thermoplastic properties, attracting considerable attention as sustainable alternatives to fossil-based plastics. Among these, poly[(R)-3-hydroxybutyrate] (PHB) has been most extensively investigated; however, its practical utility is restricted by high crystallinity and inherent brittleness, which limit its processability and range of applications [9]. To overcome these shortcomings, copolymerization has emerged as a widely explored strategy, with poly[(R)-3-hydroxybutyrate-co^®^3-hydroxyvalerate] (PHBV) being the most prominent example. PHBV exhibits superior thermomechanical properties compared to PHB, offering greater flexibility and decreased crystallinity, thereby widening its potential for industrial applications [15]. Typically, PHBV is produced by supplementing fermentation processes with structurally related carboxylic acids such as propionic, valeric, or levulinic acid [5, 7].

Despite this advantage, the use of volatile fatty acids (VFAs) as substrates poses major bioprocess challenges, primarily due to their inhibitory effects on microbial growth and metabolism [2, 19, 29]. Two main reason are widely recognized as central to VFA toxicity: hydrophobicity and weak acid dissociation behavior [11, 18, 27]. With increasing hydrophobicity, VFAs destabilize and permeabilize the cytoplasmic membrane [33], leading to leakage of essential ions and metabolites and thereby impairing cell viability. Moreover, as weak acids, VFAs exist in an equilibrium between dissociated and undissociated forms as described by the Henderson-Hasselbalch equation [28]. Only the undissociated molecules diffuse passively across the membrane [23]; once inside the neutral cytoplasmic environment, they dissociate, releasing protons and lowering the intracellular pH. To counteract this acidification and maintain pH homeostasis, cells actively export protons via ATP-driven transport systems, most notably H^+^/K^+^-ATPases. This process imposes a substantial energetic burden, as it not only prolongs lag phases while cells adjust to acid stress but also diverts substrate away from growth and polymer synthesis toward maintenance metabolism [18]. Collectively, these mechanisms reduce biomass formation efficiency, slow PHA accumulation, and limit overall process productivity.

While Shimizu et al., [25] demonstrated the effect of the external pH in combination with butyrate on microbial physiology, and other studies investigated different individual VFAs [14, 28], the combined effects of mixed VFAs under controlled pH conditions are yet to be shown. Thus, although the toxic effect of weak acids is well known, PHA production from VFAs is still mainly carried out at controlled pH values ~pH 7 [13, 17, 21, 32]. However, these were formerly optimised on other carbon sources like hydrogen or sugars [24, 26],or do not control pH during the process and thus are not applicable in investigating this effect [3, 20, 31].

This represents a critical knowledge gap, given that VFAs are generally available as complex mixtures in waste valorization processes such as anaerobic digestion, dark fermentation, or hydrothermal treatment of biowaste. Their abundance and low cost make them highly attractive feedstocks for bioplastic production, but overcoming the limitations imposed by their inhibitory properties is essential for enabling efficient and scalable bioprocesses.

*Cupriavidus necator H16*, formerly classified as *Wautersia eutropha*, *Ralstonia eutropha*, *Hydrogenomonas eutropha*, or *Alcaligenes eutrophus*, is a model organism and industrially relevant PHA producer that has been extensively employed for PHBV production due to its high metabolic versatility and ability to accumulate large intracellular PHA concentrations. Furthermore, *C. necator H 16* has been found to utilize a variety of VFAs by several researchers [13, 16, 19]. Its robust physiology and flexible substrate utilization make it particularly suitable for processes employing VFAs as carbon sources, as reported in our previous study [19]. However, the interplay between extracellular pH, VFA toxicity, and PHBV synthesis in this strain remains poorly understood. Addressing this gap is critical for optimizing process performance and ensuring that waste-derived VFAs can be harnessed effectively for sustainable copolymer production. In this context, we expect that extracellular pH strongly influences the extent of acid toxicity and, consequently, microbial performance during VFA-driven fermentations. As VFAs are increasingly present in their dissociated form at pH > 7.0 (which are expected to display a lower ability to permeate the cell membrane), we further anticipate an improvement of biomass and PHBV yields. Based on this rationale, this study aims to systematically assess the influence of VFA concentration on growth, quantify the effects of pH on biomass and PHBV yields, and determine optimal conditions for maximizing biomass and copolymer production in *C. necator H16* using a mixture of various VFAs.

## Methods

### Microbial strain

The microorganism used in this study was *Cupriavidus necator H16 (DSM 428)*. The strain was purchased from the German Collection of Microorganisms and Cell Cultures (DSMZ). For long term storage the strain was cryopreserved at -80 °C on 16.5% glycerol (Applichem).

For cultivation, the microorganisms were streaked on LB agar plates, which were stored at 4 °C and used within 3 weeks.

### Inoculum preparation

For the first seed culture, *C. necator H16* was aseptically transferred from agar plates into 1 mL reaction tubes with LB medium with 10 g·L^-1^ tryptone (Bacto, Pancreatic Digest of Casein), 5 g·L^-1^ yeast extract (Leiber, Fermentation E), 5 g·L^-1^ sodium chloride (ChemSolute). The culture was thoroughly mixed in order to homogenize the cell lumps from the agar plate culture and transferred into a 100 mL baffled shake flask containing 20 mL LB medium. The shake flask was incubated at 120 rpm and 30 °C in an incubator shaker (Infors HT, Switzerland).

After 8–12 h, at exponential phase, the culture was inoculated to the second seed culture with an optical density (OD_600_) of 0.1 in a modified Schlegel mineral salt medium (MSM). The MSM was composed of 10 g·L^-1^ Fructose (Applichem), 4.5 g·L^-1^ Na_2_HPO_4_ (Merck), 1.5 g·L^-1^ KH_2_PO_4_ (Merck), 4.4 g·L^-1^ (NH_4_)_2_SO_4_ (ChemSolute), 0.05 g·L^-1^ ammonium ferric citrate (Carl Roth), 0.01 g·L^-1^ CaCl_2_ × 2H_2_O (Merck), 0.2 g·L^-1^ MgSO_4_ × 7H_2_O (Th Geyer), 1 mL/L SL6 trace element solution. SL6 solution consisted of 1 g·L^-1^ ZnSO_4_ × 7H_2_O (VWR), 0.3 g·L^-1^ MnCl_2_ × 4H_2_O (Carl Roth), 3 g·L^-1^ H_3_BO_3_ (Applichem), 4 g·L^-1^ CoCl_2_ × 6H_2_O (SigmaAldrich), 0.1 g·L^-1^ CuCl_2_ × 2H_2_O (Applichem), 0.4 g·L^-1^ NiCl_2_ × 6H_2_O (ChemSolute), 0.3 g·L^-1^ Na_2_MoO_4_ × 2 H_2_O (Carl Roth). If necessary, pH was adjusted to 7.0 with NaOH (Applichem). The preculture was incubated at 120 rpm and 30 °C in an incubator shaker for 16 h. To remove residual fructose, the inoculum was centrifuged at 4,680 g for 20 min and was resuspended in sterile 0,9% NaCl (ChemSolute). The washed biomass was then used to inoculate the bioreactor to an OD of 1. **F**or microbioreactor inoculation, the optical density (OD) of a resuspended preculture pellet was determined and adjusted to an OD of 2 with sterile 0.9% NaCl solution.

### Fermentation in micro bioreactors

For characterization of growth at different concentrations of VFA mixture, microbioreactor studies were performed. An aerated microbioreactor (Beckman Coulter, USA) equipped with automated backscatter measurement for biomass quantification was used. The medium composition was identical to that used in the seed culture, however, supplemented with VFAs instead of fructose. The VFA stock solution contained 105 g∙L^-1^ acetic acid (Carl Roth), 135 g∙L^-1^ propionic acid (SigmaAldrich), 35.5 g∙L^-1^ butyric acid (SigmaAldrich), 10.7 g∙L^-1^ iso-butyric acid (SigmaAldrich), 18.5 g∙L^-1^ valeric acid (SigmaAldrich), and 9 g∙L^-1^ iso-valeric acid (SigmaAldrich). The stock solution was added to final concentrations ranging from 0.05 to 7.5 g∙L^-1^ total VFA in the final medium. At last, pH was adjusted to 7.0 using 4 M NaOH, volume was adjusted with deionized water, and the medium was sterile filtered using a 0.2 μm filter. Into every well of the flower plate, 950 µL of the medium were aseptically transferred. An aliquot of 50 µL of the prewashed *C. necator* H16 suspension (adjusted to an OD of 2) was then used to inoculate each medium-filled well of the flower plate to reach a final OD of 0.1. Each condition was examined as a biological triplicate. Backscatter was measured every 8 min.

### Fermentation in bioreactors

Fermentation was conducted using the four parallel glass Bioreactor Systems (76SR1000ODLS, DASGIP, Germany) with a working volume of 1.0 to 1.2 L equipped with the DASGIP PH4PO4L and TC4SC4B module for control. The temperature was maintained at 30 °C. The pO_2_, measured by a Clark electrode, was set to 20% and was controlled by stirrer speed (200 rpm to 1100) with an aeration of 1 vvm. To evaluate the effect of pH, fermentation was performed at pH 6, 6.5, 7.0, 7.5, 7.75, 8.0 and 8.5.

**Fig. 1:**
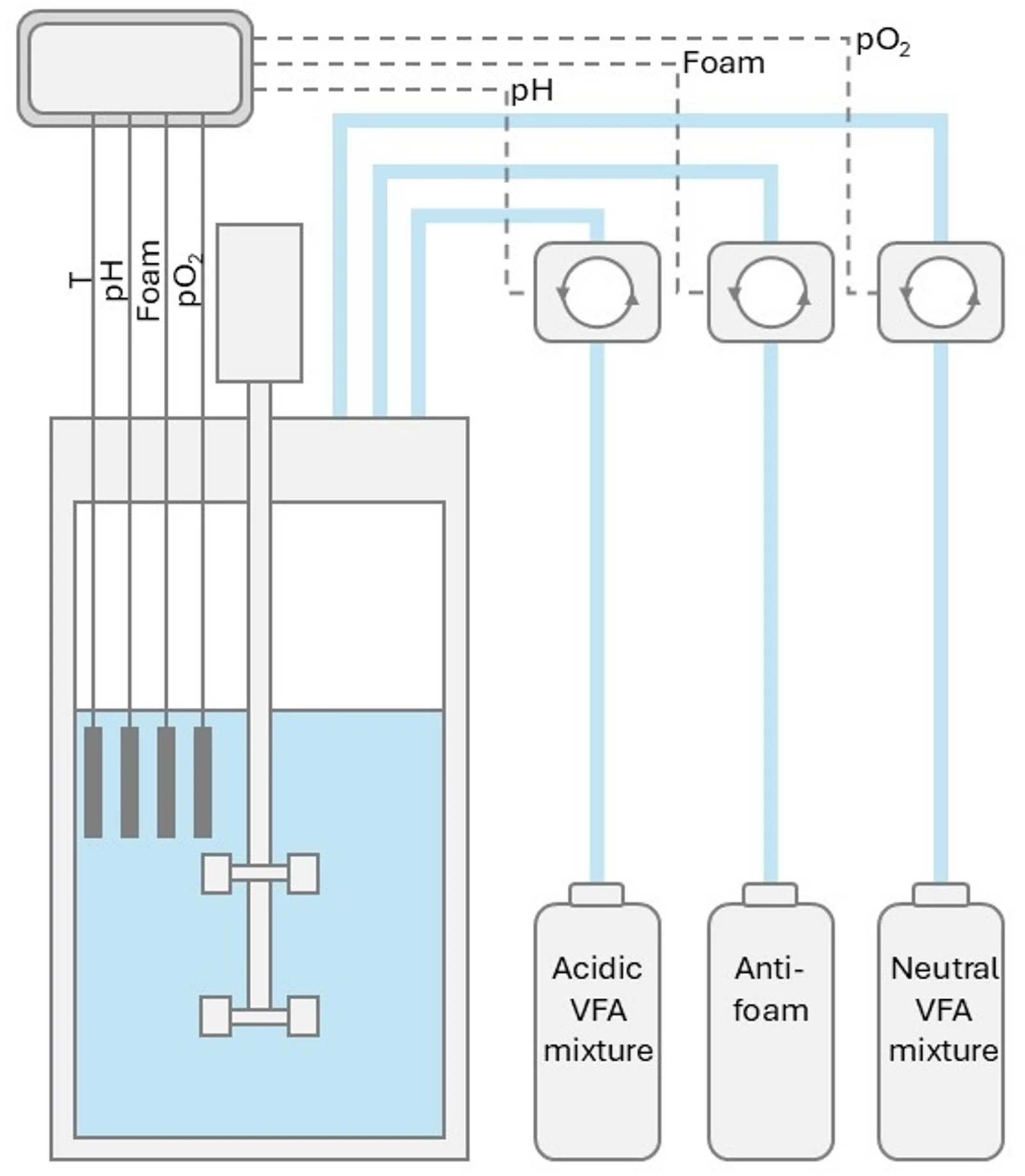
pH stat feeding strategy with pO_2_ pulses for continuous addition based on VFA consumption

To maintain a target VFA concentration of 0.5 g∙L^-1^*c*_*VFA, total*_, substrate feeding was controlled via a pH-stat approach. This method, commonly applied for organic acids, utilizes the acidic carbon source for pH adjustment, thereby theoretically ensuring that substrate addition reflects the amount consumed by the cells during fermentation ([13]; [1, 25]). Here, the pH was controlled using a VFA stock solution, a described above. The feed stream used in this work was a synthetic substrate, resembling a 50x concentrate of acidic hydrolysate, according to Prakash et al., [22].

Without counteracting effects, the VFA from the pH-dependent feeding would replace all metabolized VFA. However, the consumption of NH_4_^+^ resulted in the acidification of the culture broth, which continuously titrated against VFA uptake. Consequently, the concentration of VFAs in the culture broth consistently decreased. Upon depletion of the VFAs, the oxygen uptake rate declined rapidly, resulting in a subsequent spike in pO_2_ levels. As suggested by Huschner et al., [13], a pO_2_-dependent bolus feed was introduced once the pO_2_ signal exceeded 25% saturation. The pO_2_-dependent feed used the same VFA stock, as described above, but was neutralized to pH of 6.5 to avoid strong pH fluctuations upon the addition of the bolus. The fed volume was set to achieve a total VFA concentration of 0.5 g∙L^-1^ in the bioreactor culture. As the pH dependent feed can only replace the existing VFA, we assumed that the size of the pO_2_-dependent bolus feed roughly determined the average concentration of VFAs in the culture medium, by setting an upper limit. Increasing the size of the bolus feed would thus increase the average VFA concentration during the cultivation time. The size of the bolus feed was chosen by setting an upper limit including a safety margin below the limiting VFA concentration determined in the previous microbioreactor experiments. This pH-pO_2_-stat method, as presented in Fig. 1, allows controlled feeding without risk of inhibiting cellular growth with increased VFA-levels due to accumulation. The published Huschner et al., [13] protocol, found a C: N of 5 during growth phase to be optimal on VFA. As this study aimed to evaluate the effect of pH on the efficiency of carbon and nitrogen utilization, the optimal C: N ratio of the growth feed needs to be assumed as pH-specific. Consequently, the ratio determined by Huschner et al., at pH 6.9 was not applicable to the conditions investigated here. Therefore, in contrast to the Huschner et al., [13] protocol, 10 g∙L^-1^ of ammonium sulfate were added into the batch medium instead of a constant feed of ammonium during the growth phase.

For bioreactor cultivations, the same Schlegel mineral salt medium was used, however, the carbon source was modified to 0.5 g∙L^-1^ of the VFA mixture, while the ammonium concentration was increased to 10 g∙L^-1^. Polypropylene glycol (PPG P 2000, SigmaAldrich) (10%) was used as an antifoam agent.

### Analytical procedure

Biomass concentration (*c*_*X*_) has been estimated gravimetrically by centrifuging 15–50 mL culture broth in a pre-dried and pre-weighed centrifuge tube for 20 min at 4,680 g and 4 °C (ThermoFischer, USA). The supernatant has been used for further analysis of VFAs and Ammonium. The cell pellet has been washed with 10 mL of demineralized water and centrifuged again. The centrifuge tube containing the washed cell pellet has been frozen and lyophilized (Martin Christ, Germany) for at least 4 days. The falcon tube was weighed again and c_X_ has been calculated from the mass difference, divided by the volume used. The dried cell pellets where then used for PHA quantification according to Tamang et al., [30] and analyzed in analytical duplicate using a GC-2010 Pro gas chromatograph (Shimadzu, Germany). The GC was equipped with a flame ionization detector (FID) and an SH-Polarwax column (30 m × 0.25 mm). The chromatographic separation was performed under a temperature program starting at 120 °C (held for 0.5 min), followed by a ramp (10 °C∙min^-1^) to 230 °C with a final hold of 1 min. Helium served as the carrier gas at a constant flow rate of 3 mL·min^-1^. A sample volume of 1 µL was injected with a split ratio of 1:40.

Volatile fatty acids (VFAs), including acetic acid, propionic acid, butyric acid, iso-butyric acid, valeric acid, and iso-valeric acid, were quantified from the supernatant using gas chromatography with flame ionization detection (GC-FID) equipped with SH-Polarwax column (GC-2010 plus, Shimadzu). The pH of the samples was adjusted to below pH 2 by mixing 500 µL of the sample with 500 µL of 10% phosphoric acid and 2 µL of the mixture was injected into the GC. Helium was used as a carrier gas and the temperature of the injector, and the detector was set at 240 °C. The column was initially at 100 °C and then heated to 170 °C at a rate of 10 °C.min^-1^. Each sample was measured as technical duplicate. The calibration of the GC was performed using analytical grade (99.99%) VFAs. Their VFA concentration is presented as the sum of the single volatile fatty acids.

Ammonium was quantified enzymatically using the 4BioCore analyser (4BioCell GmbH & Co. KG, Germany).

### Mass balance, determination of rates and statistical analysis

The duration of the lag phase (*λ*) and specific growth rate (*µ*_*mean*_) was estimated by linear regression according to [4].

For microbioreactor studies, apparent growth rate was calculated from *Backscatter BS* as

$$\:{\mu\:}_{App}=\frac{\mathrm{ln}\left({BS}_{i+1}\right)-\mathrm{ln}\left({BS}_{i}\right)}{{t}_{i+1}-{t}_{i}}$$

with *Backscatter*$\:{BS}_{i}$ in a.u. and *process time*$\:{t}_{i}$ in h.

To reduce noise, growth rate *µ* was shown as moving average over 10 points, which equals to a time frame of 80 min.

*PHA content* in % CDW was used to calculate the concentration of f $\:{c}_{PHA}$ in g·L^-1^ from biomass concentration $\:{c}_{X}$ in g·L^-1^

$$\:PHA\:content=\frac{{c}_{PHA}}{{c}_{X}}$$

Residual biomass *c*_*X, res*_ in g·L^-1^ was calculated as

$$\:{c}_{X,res}={c}_{x}-{c}_{PHA}$$

with total biomass $\:{c}_{x}$ and $\:{c}_{PHA}$ in g·L^-1^.

Biomass-specific production rate *q*_*p*_ in g·g^-1^·h^-1^ was calculated by

$$\:{q}_{P}=\frac{1}{{\stackrel{-}{m}}_{X,res}}\bullet\:\frac{{m}_{PHA,i+1}-{m}_{PHA,\:i}}{{t}_{i+1}-{t}_{i}}$$

with $\:{m}_{PHA,\:i}$ representing the mass of PHA and the average, residual biomass $\:{\stackrel{-}{m}}_{X,res}$ in g.

Biomass and PHA yields were ($\:{Y}_{X/VFA}$ and $\:{Y}_{X/N{H}_{4}^{+}}$) estimated using linear regression as described by Doran [8].

Variance was calculated to describe the ability of the bioreactor set up to hold parameters like pH and VFA concentration at the desired levels. Statistical errors were determined as standard errors. A one-sample, one-sided t-test was used to assess whether the mean values at pH > 7.25 (pH 7.5, 7.75, and 8) were significantly higher than the corresponding pH 7 reference value; however, this approach assumes that the pH 7 value is a fixed reference and does not account for its experimental variability. Monotonic correlation between two variable was assessed by spearman’s rank correlation to determine the strength of a relationship using the Python package ScyPy. Given the small sample size, statistical significance was tested using a two-sided permutation test based on all permutations of the observed data.

The authors used the generative AI tool ChatGPT 5.2 (OpenAI, USA) exclusively for linguistic editing, including grammar and style improvements under the strict supervision of the authors. No substantive content, analysis, or interpretation was generated by AI. This use is consistent with the recommendations of the Committee on Publication Ethics (COPE) regarding the transparent and responsible application of artificial intelligence tools in scholarly publishing.

## Results and discussion

### VFA concentration influences growth characteristics

**Fig. 2:**
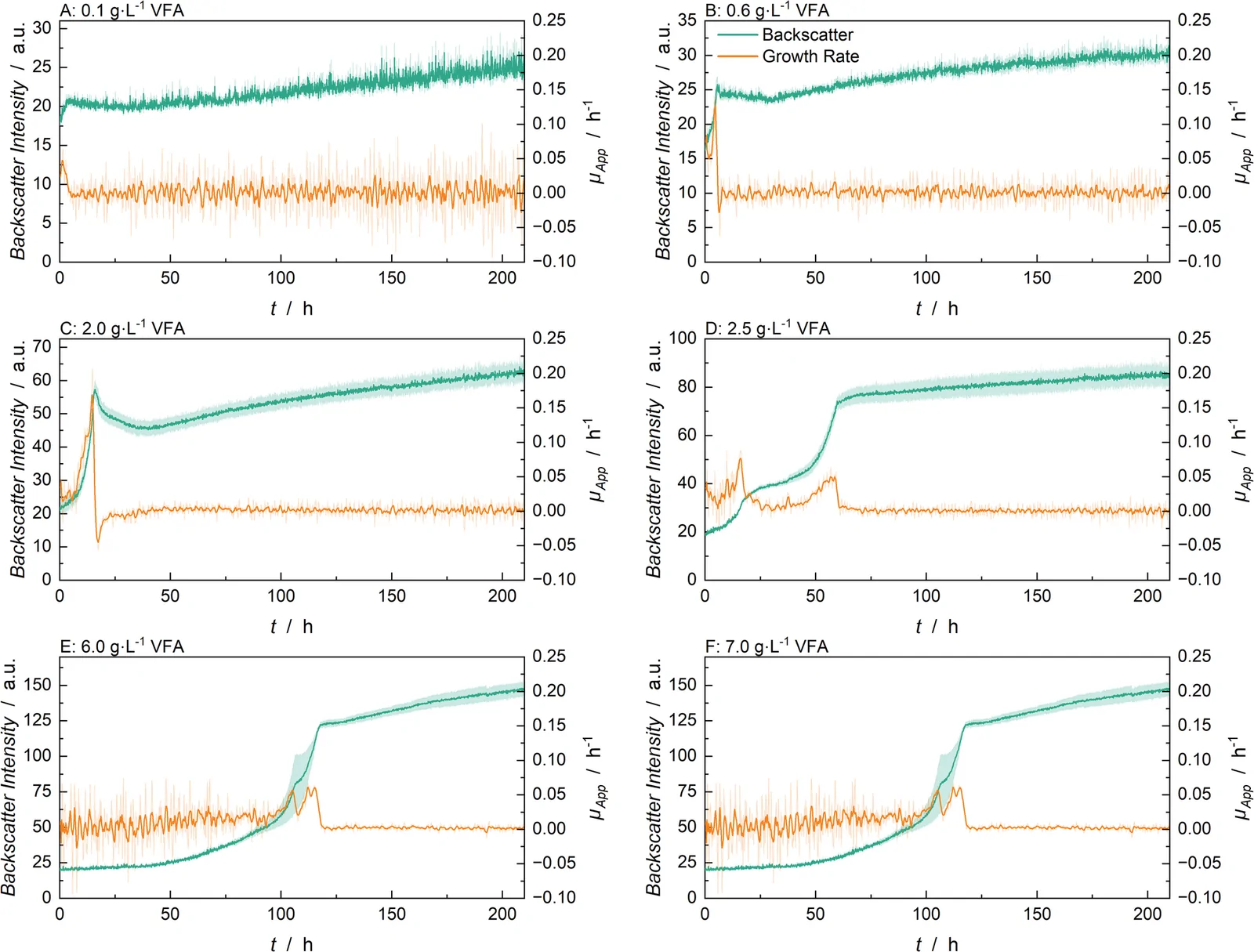
Time course of backscatter and growth rate for C. necator H16, grown on 0.05 g∙L^-1^ to 7.0 g∙L^-1^ of synthetic VFA mixture in a microbioreactor system. Raw data are represented by transparent lines, bold lines show the 20-points-moving average

**Fig. 3:**
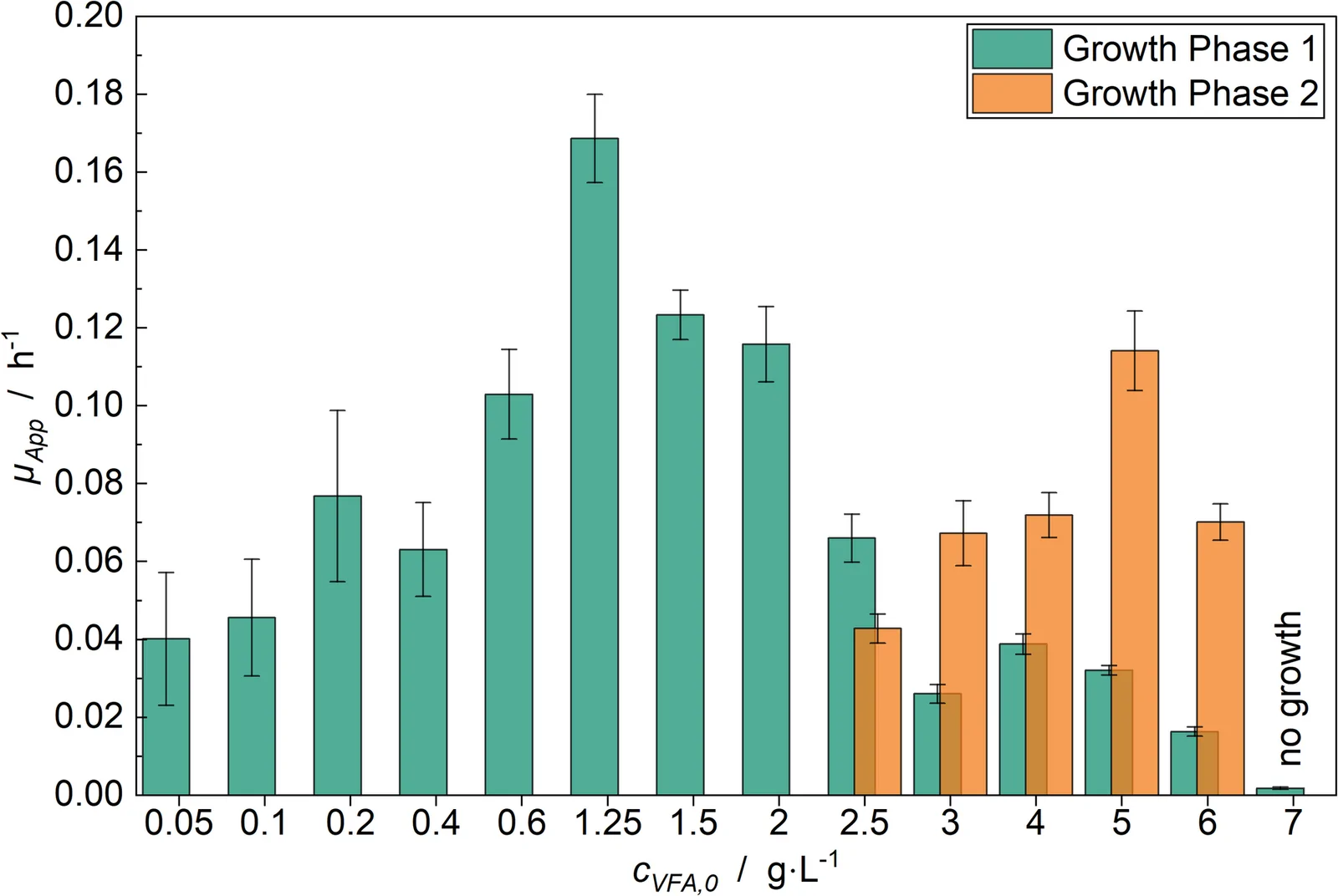
Influence of c_VFA,0_ onto growth rate

As a first step, the influence of the VFA mixture on the growth of *C. necator H16* was assessed in a microbioreactor batch system. Backscatter Intensity as a measure for biomass and specific growth rates *µ*_*App*_, determined from the respective backscatter measurements, are presented in Figs. 2 and 3, as well as in the supplementary material. The inhibitory effects reported for individual VFAs on *C. necator H16* in our previous study [19] and by Jawed et al., [16] were also observed in the present study when applying a mixture of VFAs. At initial VFA concentration *c*_*VFA,0*_ ≤ 2.0 g∙L^-1^, backscatter and growth rate indicated monophasic growth without pronounced lag phases ( Figs. 2A-C and 3). In contrast, increasing the *c*_*VFA,0*_ greater than 2.5 g∙L^-1^ resulted in multiphasic growth characterized by distinct lag phases between successive growth periods ^1^ (Fig. 2D-E). Such multiphasic behaviour may arise from carbon catabolite repression (CCR) when microorganisms are exposed to multiple carbon sources [10]. Under CCR, the uptake and metabolism of less favorable substrates are temporarily repressed in favor of preferred carbon sources, thus showing a sequential use of substrates in batch cultivations. On a metabolic level, this maybe explained due to the fact, that there is no central catabolic pathway for VFAs. In *C. necator H16*, acetate and propionate are activated to acetyl-CoA and propionyl-CoA and thus are metabolised by the citric acid cycle and respectively by the methylcitrate cycle. However, longer chain VFAs like butyrate or valerate are primarily metabolised through β-oxidation, generating acetyl-CoA and, in the case of odd-chain VFAs, propionyl-CoA [34]. Furthermore, a recent study published by Holmes et al., [12] demonstrated that C. necator exhibits VFA- and chain-length-dependent expression of genes involved in VFA catabolism, indicating that utilisation of individual VFAs may require distinct metabolic responses. In a medium containing a VFA mixture as carbon source, a transition between substrates may thus require an induction or adjustment of the corresponding metabolic pathway ultimately leading to a temporarily reduction on growth rate during this adaption period. Thereby, the observed multiphasic growth behaviour is displayed. While the sequential use of VFAs has not been further studied in this work, Yun et al., [35] observed this behaviour in a cultivation using a mixed VFA carbon source, thus supporting the interpretation that different VFAs may not be consumed simultaneously.

In fed-batch systems, this can lead to transient substrate accumulation, which in the case of VFAs is particularly detrimental due to their toxic effects. Supporting this, a study conducted by Chakraborty et al., [6] found an accumulation of certain VFAs at higher concentrations in a continuously fed bioprocessing strategy. Following this interpretation, suppression of multiphasic growth is essential for stable and efficient process operation and was therefore a key requirement in this study.

At the concentration ≥ 7.0 g∙L^-1^ of VFA mixture, no growth was observed (Fig. 2F). The mixture consisted mainly of acetic and propionic acid, for which inhibitory concentrations above 10 g∙L^-1^ have been reported individually on our previous study [19]. Remarkably, complete growth inhibition was already observed at 7.0 g∙L^-1^ for the VFA mixture, which is comparable to inhibition levels reported for valeric acid alone [19]. This finding indicates that combined VFAs exert a stronger toxic effect than the corresponding single acids, suggesting additive or synergistic inhibition mechanism. However, a lag phase increasing with VFA concentration has been observed, with a lag phase of 44 ± 2 h at 6.0 g∙L^-1^ VFA. If growth would happen at 7.0 g∙L^-1^ with an lag phase > 220 h (the observed cultivation time) we would not have detected it in this set up, providing a critical limitation.

The highest specific growth rate *µ*_*App*_ was observed at *c*_*VFA,0*_ = 1.25 g∙L^-1^ (See Figs. 2 and 3), while the concentrations higher or lower than this value resulted in a reduced growth rate. Furthermore, for *C*_*VFA,0*_ ≥ 3.0 g∙L^-1^, *µ*_*App*_ during the first growth phase was significantly lower than during the second phase (Fig. 3). This behaviour may be attributed to a gradual reduction in inhibitory concentration as the total VFA concentration decreased over time during batch cultivation, thereby allowing partial recovery of metabolic activity in the later growth phase.

### Bioreactor operation with pH-pO2-stat fed-batch process

**Fig. 4:**
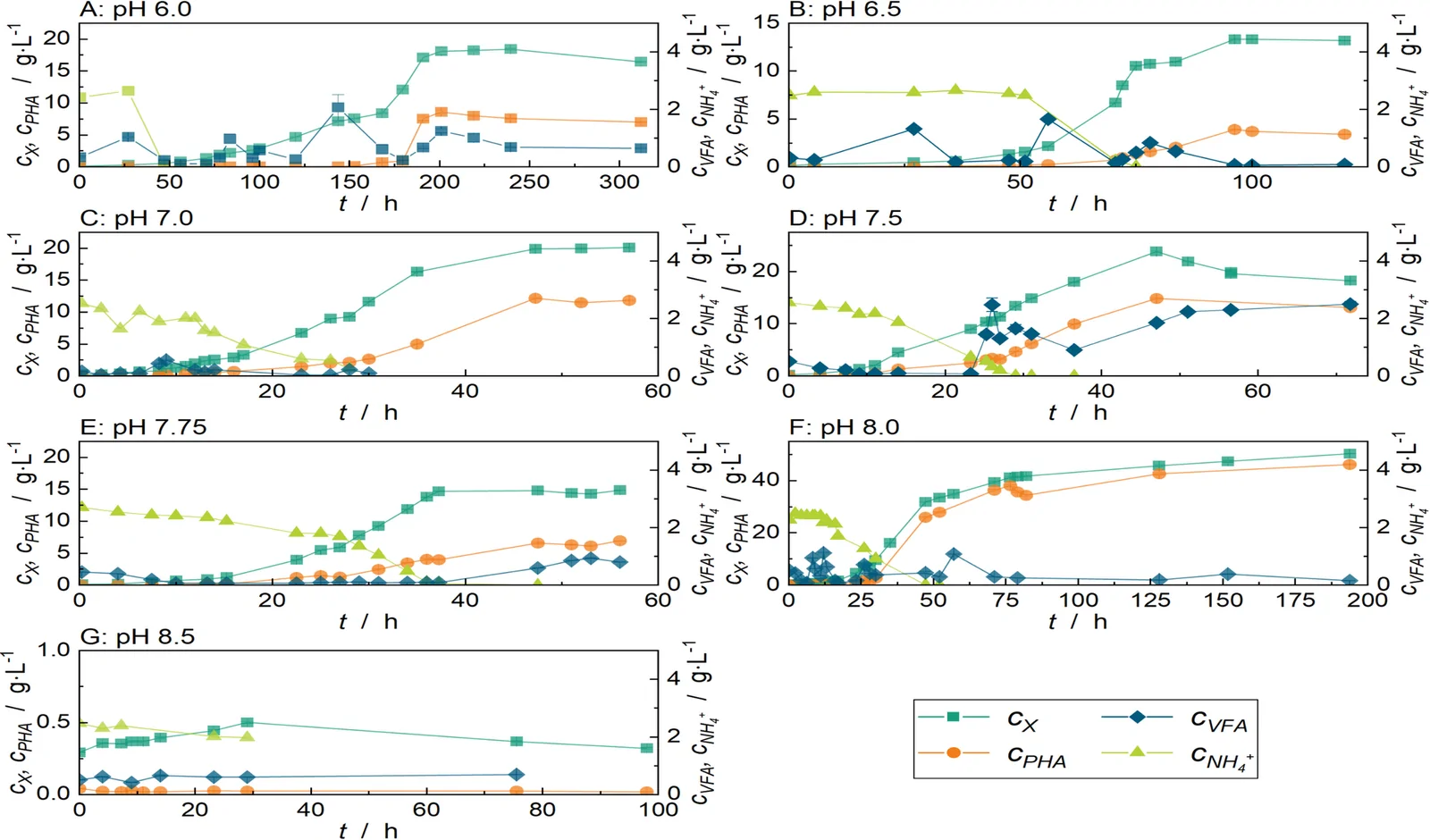
Concentrations of total biomass, PHA, NH_4_^+^ and VFA cultivating C. necator H16 on VFA at pH 6.0 to 8.5

As observed in the preceding experiments, VFA concentration was identified as a critical factor affecting growth behavior of the *C. necator H16*. Therefore, to specifically investigate the effect of pH on microbial growth and PHA production while maintaining constant substrate conditions. Figure 4 shows the growth and production profile of the fed batch experiment performed at pH values ranging from 6.0 to 8.5.

During the fed batch experiments, the concentrated VFA mixture was fed using a pH-stat with pO_2_ pulses method as previously described. Although, an optimum VFA concentration of 1.25 g∙L^-1^ resulted the highest specific growth rate (Fig. 3), a conservative target concentration (*c*_*VFA*_, _target_) of 0.5 g∙L^-1^ was selected for bioreactor experiments. This choice was made for two main reasons: first, to compensate for potential substrate concentration fluctuations arising from the inherent response delay of the PI controller, and second, to prevent multiphasic growth by ensuring that the reactor VFA concentration remained below the critical threshold of 2.0 g∙L^-1^.

Table 1 summarizes corresponding the maximum and mean parameters in the bioreactor cultivation, underlining that the design decisions of the experiments led to a process without critical accumulation of VFA. Overall, the applied feeding strategy successfully maintained stable pH conditions and a VFA concentration close to the target value of 0.5 g∙L^-1^ for all experiments except at pH 7.5 (Fig. 4). Despite the controlled feeding approach, the VFA concentration at pH 7.5 temporarily exceeded the critical threshold of 2.0 g∙L^-1^ for less than 2 h at approximately 26 h of cultivation (Fig. 3D). This transient increase did not severely impair the growth performance, as the VFA concentration remained above the critical level only toward the end of the growth phase. However, a prolonged accumulation of VFAs was observed over a period of approximately 50 h at pH 7.5. The *c*_*VFA*_, _mean_ except for pH 7.5, remained between 0.2 and 0.6 g∙L^-1^ and *c*_*VFA* max_ reached up to 1.34 g∙L^-1^, which below the critical concentration Table 1.

**Table 1:** Key factors affecting microbial growth and polyhydroxyalkanoate (PHA) production from volatile fatty acids (VFAs). Achievement of the target VFA concentration (0.5 g∙L^-1^) and stability of pH control using a pO₂-pH-stat with concentrated VFA solute. (a) Errors represent the standard deviation as a measure of VFA and pH variability, while (b) Concentration of biomass at the point of ammonium depletion with standard errors of a technical duplicate and concentration of PHA at the end of production phase. (c) No distinct growth and production phase could be estimated for pH 8.5

pH_target_	6.0	6.5	7.0	7.5	7.75	8.0	8.5
*pH*_mean_^a)^	6.00 ± 0.04	6.50 ± 0.02	7.01 ± 0.05	7.5 ± 0.2	7.75 ± 0.04	8.00 ± 0.03	8.51 ± 0.02
*f / %*	6.20	2.05	0.66	0.21	0.12	0.07	0.02
*c*_*VFA*_, _target_ / g∙L^-1^	0.5						
*c*_*VFA*, mean_ / g∙L^-1 a)^	0.6 ± 0.5	0.4 ± 0.5	0.2 ± 0.2	0.9 ± 0.8	0.5 ± 0.5	0.4 ± 0.3	0.6 ± 0.1
*c*_*VFA, max*_ / g∙L^-1 b)^	1.25 ± 0.07	1.34 ± 0.04	0.55 ± 0.02	2.47 ± 0.8	0.93 ± 0.01	1.11 ± 0.03	0.69 ± 0.01
*c*_*X, a*_ / g∙L^-1 b*)*^	0.5	6.7	6.7	8.9	7.8	7.3	0.5^c)^
*c*_*PHA*_ / g∙L^-1 b)^	8.6	3.9	12.2	14.8	12.7	42.5	0.02

pH_target_: Target pH of the cultivation, pH_mean_: Average pH in the cultivation ± 1 SD, f: molar fraction of undissociated VFA at a pKa of 4.82, c_VFA, mean_: Average VFA concentration in the cultivation ± 1 SD, c_VFA, max_: maximum observed VFA concentration during VFA concentration, c_X, a_: Total biomass concentration at ammonium depletion, c_PHA_: final PHA concentraion

### Fermentation profile

The fermentation process is distinguished into growth and production phase. Typically, the fermentation time during the presence of ammonium indicates the growth phase where biomass is built and absence of ammonium defines the typical production phase. However, Fig. 4 shows that small amounts of PHA were produced, although total depletion of ammonium was not reached yet. A comparison to Huschner et al., could not be made, as ammonium concentrations were not published in the optimized process [13]. Yet, this leads to the conclusion, that some other component of the medium or process parameter was limiting and therefore enabling PHA production. Thus, optimization of the medium and process parmeters could lead to more efficient production of biomass in fed-batch mode.

Biomass growth and PHA production was found at pH 6.0 to pH 8.0 (Fig. 4A-F). In contrast to pH 6.0 to 8.0, no growth or PHA production was observed at pH 8.5 (Fig. 4G). Although, no significant growth was observed at pH 8.5, the cultivation showed an uptake of NH_4_^+^ (Fig. 4G). The effect led to a demand of 30.9 g of 4 M NaOH for pH correction.

#### Influence of pH onto microbial growth

**Fig. 5:**
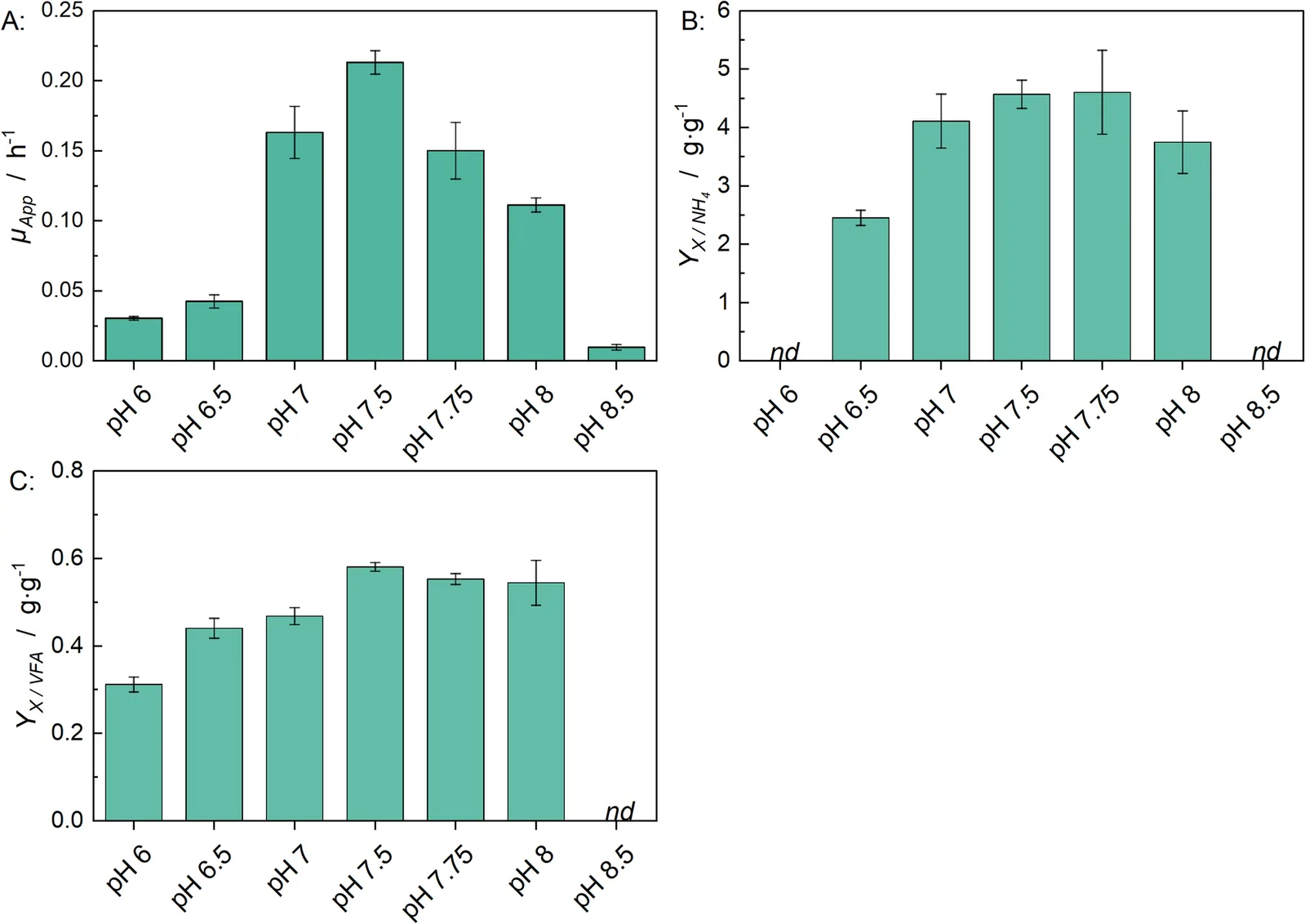
pH influence on growth rates (**A**), biomass yield on VFA (**B**) and ammonium (**C**) of C. necator on VFA due to weak acid toxicity (nd: not determined)

The effect of pH on *C. necator H16* growth on volatile fatty acids (VFAs) was evaluated using the specific growth rates (*µ*_*App*_), and biomass yield (see Fig. 5).

The *C. necator H16* showed a highest *µ*_*App*_ at pH 7.5 of 0.21 ± 0.01 h^-1^ with a biomass concentration of 8.9 g∙L^-1^ when the ammonium was completely depleted. Quantitatively, the *µ*_*App*_ at pH 7.5 is 31% higher than pH 7.0 (*µ*_*App*_ = 0.16 ± 0.02 h^-1^) and 41% higher than at pH 7.75 (*µ*_*App*_ = 0.15 ± 0.02 h^-1^). In contrast, no growth was observed at pH 8.5 and growth was strongly inhibited at pH 6.0, where only 0.5 g·L^-1^ biomass was detected at the end of the growth phase.

Notably, at pH 6.0, ammonium was completely consumed within 48 h (Fig. 4A), biomass growth continued until 168 h post inoculation, reaching a final concentration of 7.6 g∙L^-1^.This observation indicates, that ammonium may be converted into a different substance, not measured with this method As shown in Table 1, the biomass concentrations reached at the end of the growth phase at this pH values were consistent with those obtained at pH values between 6.5 and 8.0. The only exception was observed at pH 7.5, where a higher final biomass concentration was measured. However, this may be attributed to varying sampling resolution during cultivation rather than to a true difference in biomass production.

As shown in Fig. 5, conducting growth experiments at pH 6.5 or lower drastically reduces growth rates on *C. necator*. Similarly to the growth rates, the highest biomass yield (*Y*_*X/VFA*_) of 0.58 ± 0.01 g∙g^-1^ was found at a pH of 7.5. Therefore, increasing *Y*_*X/VFA*_ by 24% compared to pH 7.0 (0.47 ± 0.02). Further increasing pH to 8 slightly decreases *Y*_*X/VFA*_ (0.54 ± 0.05 g∙g^-1^) only within the error margin. *Y*_*X/VFA*_ was significantly (*p* = 0.007) increased at pH 7.5 to 8.0 compare to the commonly applied pH 7.0 using a one sample t-test. In contrast, using substrates that do not exert weak acid toxicity, the pH optimum has previously been observed at pH < 7. For example under autotrophic conditions, the pH optimum of *C. necator* was reported to lie between pH 6.4 and 6.8 [24]; Song et al., [26] observed an optimal pH at 6.9 on fructose. Therefore, microbial growth has been widely conducted at slightly acidic pH for *C. necator* since then, even when weak acids like VFAs are present [13, 16]. Thus, the results presented in this work support the initial hypothesis that increasing the pH from slightly acidic conditions to values above 7.0 enhances biomass yield and growth rate by accounting in the toxic effect of weak acids.

The biomass yield from ammonium only changed within the error margins at pH 7.0 to 8.0. The cultivation at pH 6.5 led to a reduced *Y*_*X/NH4*_ of 2.5 ± 0.1 g∙g^-1^, while the *Y*_*X/NH4*_ at pH 7.5 was 4.6 ± 0.3 g∙g^-1^. *Y*_*X/NH4*_ could not be determined for cultivations at pH 6.0 and 8.5, as no growth was observed at pH 8.5. At pH 6.0 ammonium consumption was observed time-independent of growth thus we were not able to directly correlate biomass growth and ammonium uptake.

To approximate the acid–base behavior of the VFA mixture, a weighted mean pKa was calculated based on the molar fraction of each individual VFA. Although the pKa values of the individual VFAs ranged from 4.76 to 4.87, the resulting weighted mean pKa of 4.82 provides a reasonable simplification for the mixture. Based on the Henderson–Hasselbalch equation, the theoretical fraction of undissociated VFA was estimated to be 6.2 mol% at pH 6.0, decreasing to only 0.07 mol% at pH 8.0. Thus, increasing the pH from 6.0 to 8.0 is expected to reduce the fraction of undissociated VFA by approximately two orders of magnitude, highlighting the pronounced shift toward the dissociated VFA species under alkaline conditions.

Thus, the data in Fig. 5; Table 1 support the initial hypothesis that increasing the pH increases the efficiency of biomass production.

#### Influence of the pH onto PHA production

Analogous, the influence of pH onto PHA production of *C. necator H16* from VFA was observed. Cultivating *C. necator* at pH 7.0 led to a PHA *Y*_*PHA/VFA*_ of 0.44 ± 0.06 g∙g^-1^. By increasing the pH to 8 the yield was enhanced by 19% (*Y*_*PHA/VFA*_ of 0.53 ± 0.03 g∙g^-1^). It has to me mentioned that the difference in the PHA yields at pH values of 7.5 and 7.75 were within the error margin (*Y*_*PHA/VFA*_ of 0.51 ± 0.02 g∙g^-1^ and 0.53 ± 0.2 g∙g^-1^), therefore increasing the pH to > 7.5 leads to an enhanced usage of VFA in PHA production. In contrast, by lowering the pH to 6.0 the PHA yield was decreased to 0.30 ± 0.08 g∙g^-1^. No yield could be determined a pH 8.5 as no microbial growth was observed. Using a one sample t-test we found that *Y*_*PHA/VFA*_ was significantly (*p* = 0.005) higher at pH 7.5-8.0 (*n* = 3) than at pH 7.0 (*n* = 1). Our findings are in agreement with Shimizu et al., [25], who also found the highest *Y*_*PHA/VFA*_ at pH values of 8.0, when cultivating *C. necator H16* on butyric acid. While Shimizu reported *Y*_*PHA/VFA*_ of 0.85 g∙g^-1^ on butyric acid at this pH, we observed an 0.53 ± 0.03 g∙g^-1^ on VFA mixture. Several reasons might lead to the lower yield: First, the biosynthesis of PHA from butyric acid requires the incorporation of 1 mol of H_2_O per mole of butyric acid, therefore increasing the *Y*_*PHA/VFA*_ in w/w (but not the molar *Y*_*PHA/VFA*_). This does not happen during the conversion of acetic acid or propionic acid [34]. And second, for *C. necator* the biosynthesis of PHA from iso-butyric acid and iso-valeric acid as part of the mixture used, has not been researched yet to our knowledge. Although no accumulation of these iso-acids was observed in our experiments, a conversion to PHA cannot definitely be confirmed by the methods used in this work. Both effects might lead to the difference in yields observed in these studies.

**Fig. 6:**
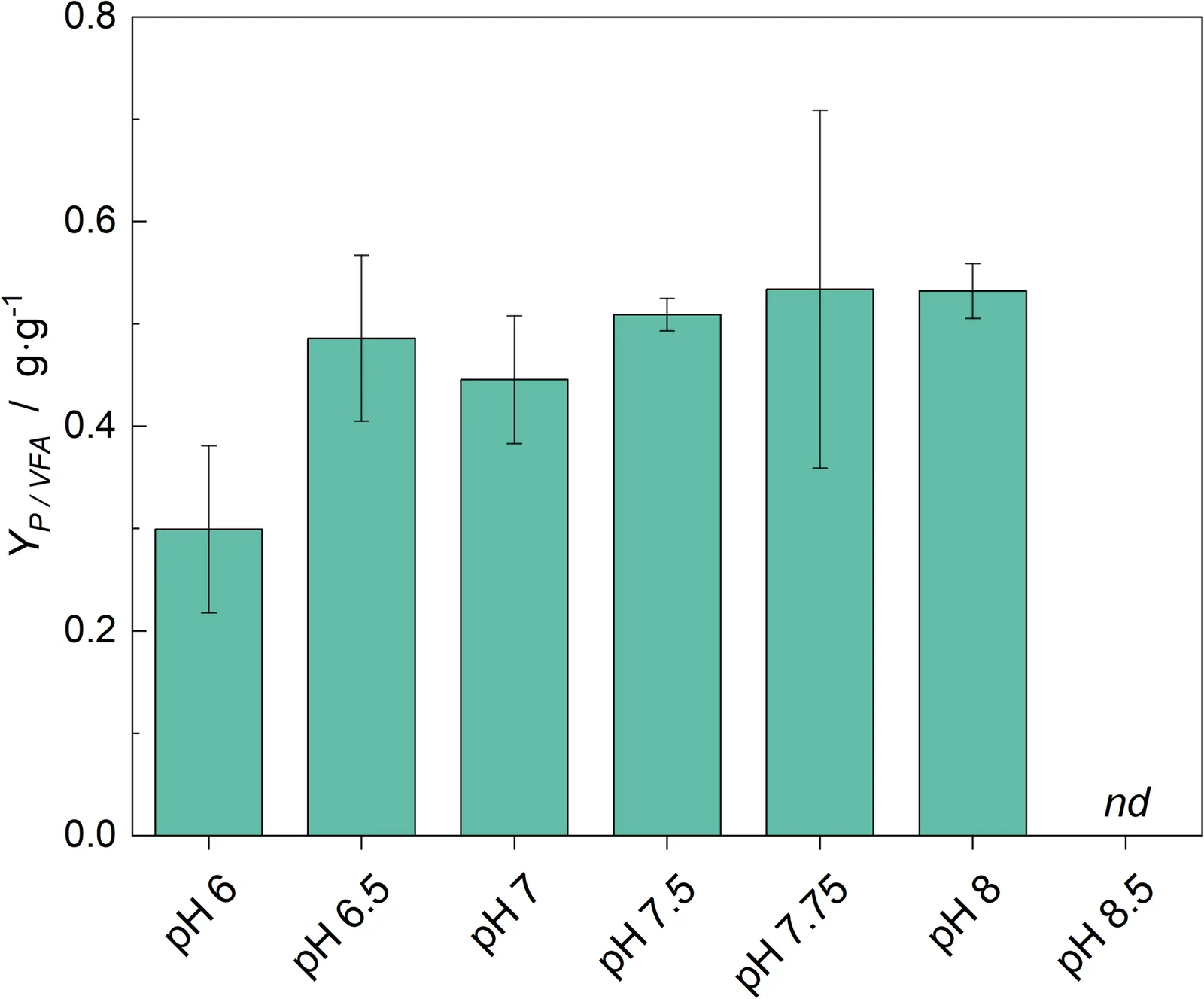
pH influence on PHA yield Y_PHA/S_ of C. necator on VFA due to weak acid toxicity

Analogous to biomass yield, the data in Fig. 6; Table 1 support the initial hypothesis that increasing the pH increases the efficiency of PHA production.

**Fig. 7:**
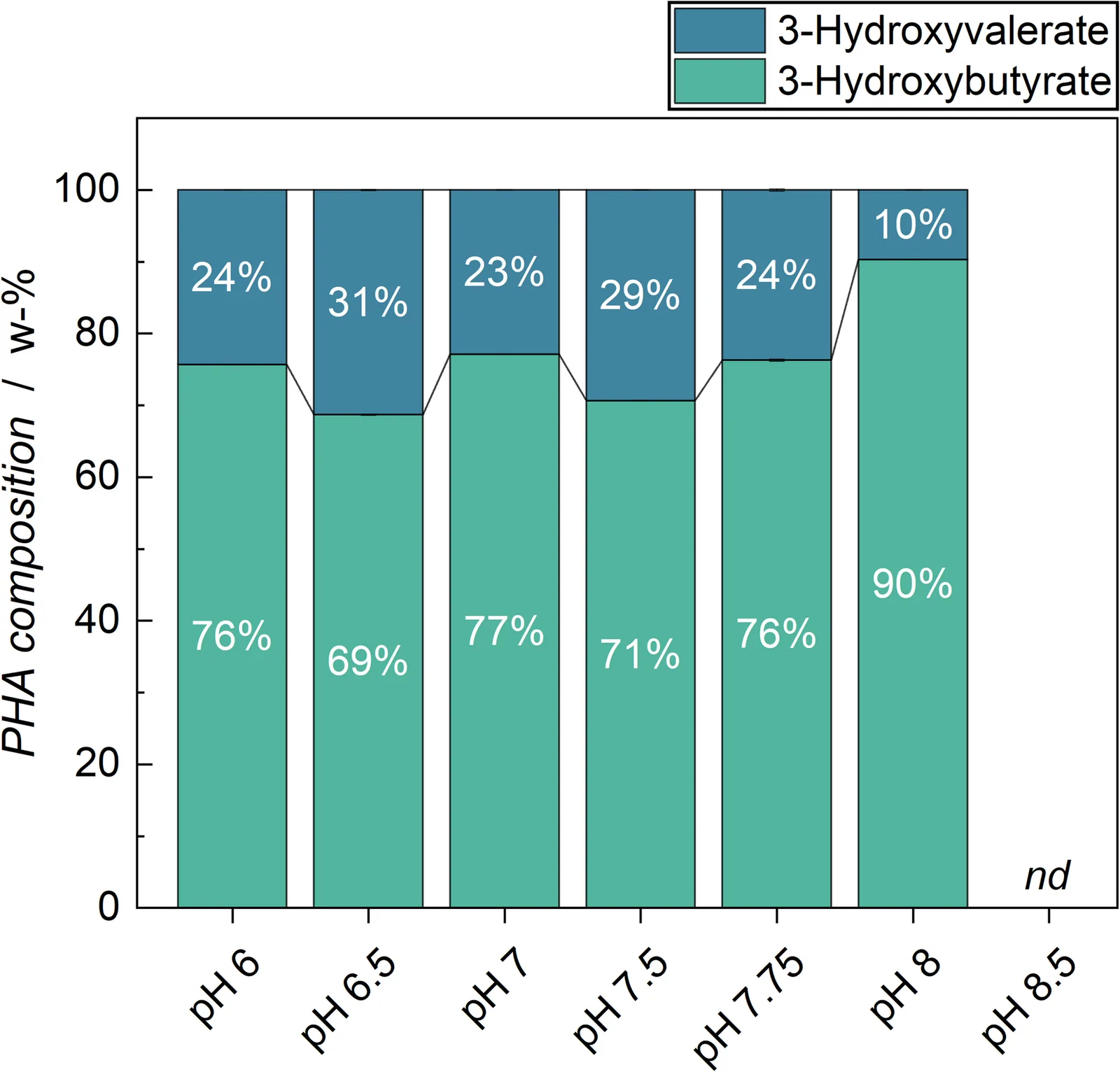
PHA composition in C. necator cultivated under different pH conditions

As the individual VFAs in the mixtures show similar, yet different pKa values, they are also differently dissociated at each pH value. Since these differences would lead to differing membrane permeabilities, the biological availability of the individual acids may therefore change across the tested pHs. This differing composition of available substrate could influence the polymer composition, as it was shown by Cai et al., [5], that the substrate composition is the major factor in the polymer composition [5]. Therefore, we tested if pH influences PHA composition using a VFA mixture. However, although 3-HV content varied between 23% and 31% for pH values of 6.0 to 7.75, no statistically significant (*p* = 0.242, *n* = 6) relationship was found between pH and 3-HV content (see Fig. 7). Nevertheless, the 3-HV content at pH 8 was notably lower than in the other cultivation conditions. Similarly, no relationship between 3-HV content and effective *c*_*VFA*_ was found.

#### Influence of the pH on biomass specific production rate

**Fig. 8:**
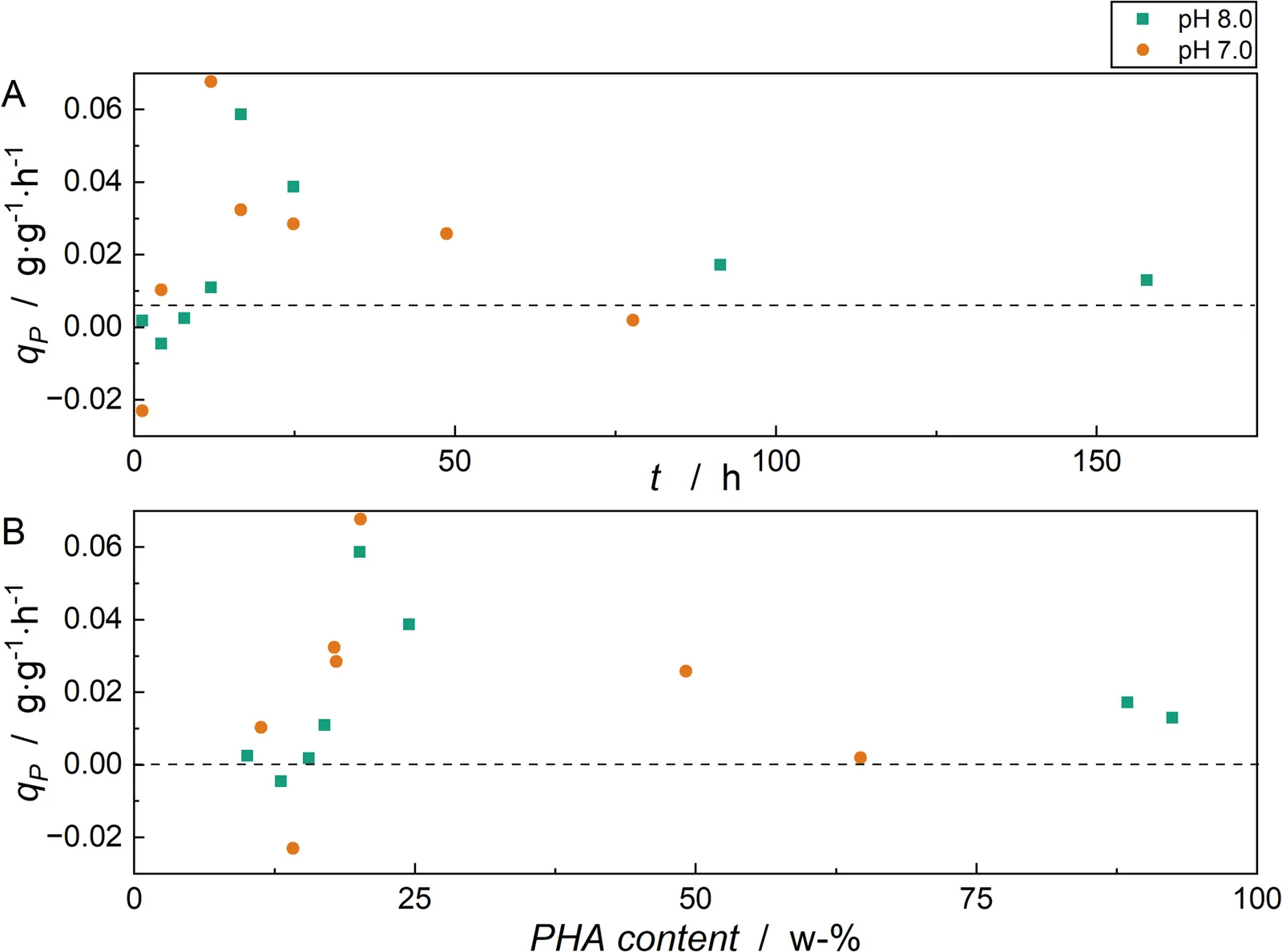
Biomass specific production rate as a function of A: time and B: PHA content

In this study we observed that biomass specific production rate *q*_*P*_ decreased during the course of the cultivations. Representatively the courses of *q*_*P*_ are shown in Fig. 8-A for cultivations at the commonly used pH 7.0 and pH 8.0 showing the highest PHA production. Production rates decreased much faster at pH 7.0 compared to cultivations at pH 8.0 leading to lower a PHA concentrations in the culture broth and per cell mass. This hints that cell mass may stay more active at higher pHs during nitrogen limited production phase, which in turn might be attributed to the lower toxicity exerted by the VFAs.

Next, the influence of PHA content in the cell onto *q*_*P*_ was observed Fig. 8-B. We found that there might be a correlation between the decreasing of *q*_*P*_ and the increasing of PHA content. However, it remains unclear whether this effect is caused by reduced cellular mobility due to PHA accumulation in the bacterium. It may instead be attributed to other inhibitory factors during cultivation, suchdecreased cell viability due to nutrient limitation.

## Conclusion

In this work we investigated the toxic effect of a weak acidic mixture to enhance microbial growth and PHA production from volatile fatty acids (VFAs). We showed that pH-stat with pO_2_-triggert pulses is a suitable feeding strategy to keep the VFA concentration below critical limits of biphasic growth. The feeding strategy led to low variability of VFA concentration within the cultivations.

The results further identify pH as an important process parameter for biomass growth and PHA accumulation in *C. necator H16* using VFAs as carbon source. Increasing the pH during growth phase from traditionally used 7.0 to 7.5 increased the specific growth rate by 31% and biomass yield on VFAs by 24%. During PHA accumulation, pH values of 7.5 to 8.0 significantly improved PHA yield. Thus, the data supports the initial hypothesis, that an pH of > 7.0 could increase the biomass and PHA yield compared to the commonly applied pH 7.0. Following this findings, the efficiency of the process may be enhanced by increasing the pH to 7.5 during growth phase and 8.0 during PHA production phase. The higher growth rate generally leads to shorter bioreactor occupation times as well as higher yields for biomass and PHA production lead to a reduced demand for resources, thus possibly improving the two big positions of production costs as a necessary step on the way to PHA commercialization.

This study helps closing the gap in understanding how PHA production processes on VFA is influenced by pH as part of the bioreactor microenvironment. 

## Data Availability

No datasets were generated or analysed during the current study.
